# Fluoroscopic Catheter-Tip Positioning During Internal Jugular TIVAD Placement in Breast Cancer: A Two-Center Retrospective Cohort Study

**DOI:** 10.3390/diagnostics16203310

**Published:** 2026-10-12

**Authors:** Zeyang Fan, Kai Yang

**Affiliations:** 1 Department of Interventional Radiology and Vascular Surgery, Peking University First Hospital, Beijing 100034, China; fanzeyang@pku.edu.cn; 2 Department of Interventional Radiology, The Fourth Hospital of Hebei Medical University, Shijiazhuang 050011, China

**Keywords:** breast cancer, totally implantable venous access device, internal jugular vein, fluoroscopy, catheter-tip positioning, catheter geometry, cavoatrial junction, intraprocedural correction

## Abstract

**Background/Objectives:** Fluoroscopy permits direct assessment of catheter course and tip position during totally implantable venous access device (TIVAD) placement. We compared left- and right-sided internal jugular vein (IJV) port placement in breast cancer. **Methods:** This retrospective two-center cohort included 836 ultrasound- and fluoroscopy-guided IJV procedures performed during 2021–2024. The institutional optimal radiographic band was 20–40 mm below the carina; 10–20 and 40–45 mm were adjacent acceptable bands. **Results:** Left and right IJV access were used in 478 and 358 procedures. The imaging-defined reference lengths were 25.0 ± 0.5 and 22.2 ± 0.5 cm. First-pass target success was 70.3% and 77.1%, respectively. Guidewire-assisted repositioning (9.6% vs. 2.8%) and abnormal initial catheter course (7.9% vs. 2.0%) were more frequent on the left. Final tips were in the optimal band in 97.1% and 96.6%, and in the broader acceptable band in 99.4% on each side. Immediate complications comprised three pneumothoraces and six bleeding events. Among 823 patients with documented 90-day status, 45 had a first device-related event (12 mechanical, 18 thrombotic, 10 infectious, and 5 other). **Conclusions:** Left IJV placement involved a longer route and more intraprocedural correction, while final tip-position distributions were similar. Fluoroscopic assessment documented and guided the correction sequence.

## 1. Introduction

Totally implantable venous access devices (TIVADs) provide durable access for anticancer treatment and supportive care. Contemporary vascular-access guidance addresses ultrasound-guided venipuncture, central tip placement, and image- or electrocardiography-guided confirmation for long-term devices [1]. The NAVIGATE position statement standardizes device terminology [2]. Clinical guidance and recent reviews describe port implantation and management [3,4,5,6]. For upper-body central venous catheters, current guidance favors a tip in the lower superior vena cava (SVC), at the cavoatrial junction (CAJ), or in the high right atrium [1].

Published TIVAD studies assess final tip location using radiography, CAJ landmarks, insertion-depth estimates, three-dimensional reconstruction, or intracavitary electrocardiography [7,8,9,10,11,12]. Postoperative radiography documents the final tip; intraprocedural fluoroscopy also shows proximal or distal positioning, azygos entry, kinking, and other unfavorable trajectories before fixation. These findings characterize the steps required to achieve the final position.

The left IJV route crosses the left brachiocephalic vein before reaching the SVC and is generally longer and more angulated than the right-sided route. A breast-cancer study of port migration also examined differences between right and left chest ports [13]. In a separate series of 1510 patients with breast cancer, malposition occurred more often after left than right IJV access (4.96% vs. 0.66%); catheter manipulation or digital subtraction angiography (DSA)-assisted repositioning corrected most malpositions [14]. Guidance recognizes longer approximate insertion distances from the left IJV and describes contrast fluoroscopy as a standard imaging method in technically difficult long-term access [1].

In breast-cancer care, access side is influenced by tumor laterality and prior surgery. We examined how left and right IJV access differed during fluoroscopic TIVAD positioning, with particular attention to the steps between initial and final tip assessment.

## 2. Materials and Methods

### 2.1. Study Design and Population

This retrospective two-center cohort study included consecutive adults with breast cancer who underwent IJV TIVAD placement at Peking University First Hospital and The Fourth Hospital of Hebei Medical University between January 2021 and July 2024. Eligibility required baseline and operative records, intraprocedural fluoroscopic documentation, and postoperative chest radiography sufficient for catheter-course and tip assessment. Non-IJV procedures and cases without adequate imaging were excluded. Reporting followed the STROBE statement [15]. The study was conducted in accordance with the Declaration of Helsinki and was approved by the Ethics Committee of Peking University First Hospital (PKUFH-26-301; 9 May 2026) and the Ethics Committee of The Fourth Hospital of Hebei Medical University (FMH2607-6; 3 July 2026). Written informed consent was obtained from the patients. Both ethics committees approved publication of the anonymized clinical images.

### 2.2. Port Placement and Access-Side Selection

Eight staff interventional radiologists, four at each center, independently performed the procedures. Their experience ranged from 6 to 13 years, and recorded annual TIVAD volumes ranged from 22 to 37 per operator. Venipuncture was performed under real-time ultrasound guidance, and catheter course, tip position, and adjustments were assessed with fluoroscopy. Both centers used a 6.6-F polyurethane port system. Access vein and reservoir side were routinely selected contralateral to the affected breast and prior breast or axillary surgical field.

### 2.3. Fluoroscopy-Guided Catheter-Tip Positioning and Correction

**Figure 1:**
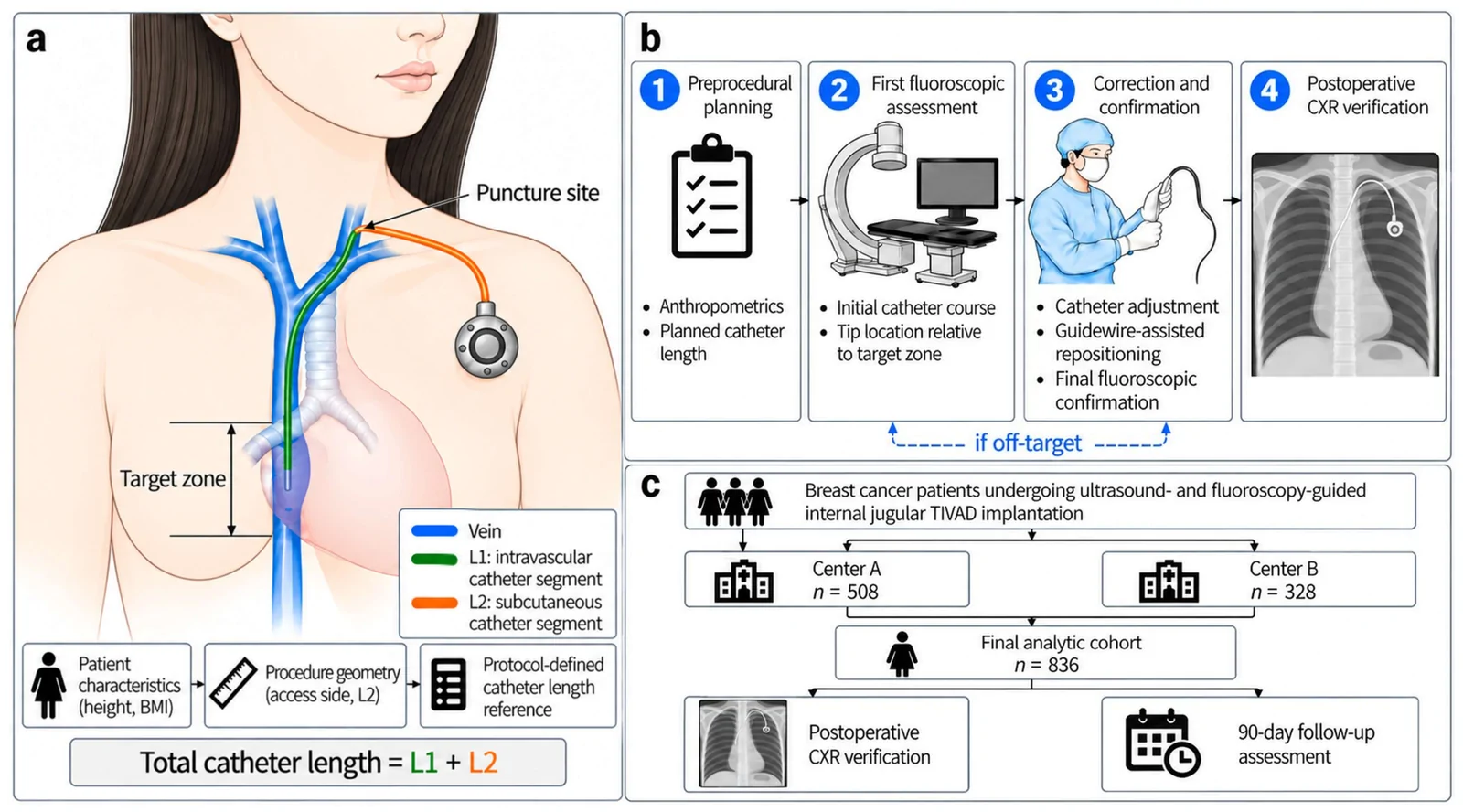
Catheter-length framework, fluoroscopic workflow, and two-center study flow. (**a**) Schematic left internal jugular vein (IJV) access and catheter segments: intravascular (L1) and subcutaneous (L2). Distances are defined in Section 2.4. (**b**) Length planning, initial fluoroscopic assessment, correction and confirmation, and postoperative chest-radiograph verification. (**c**) Two-center cohort and 90-day follow-up assessment. BMI, body mass index; CXR, chest radiograph; TIVAD, totally implantable venous access device.

The workflow comprised length planning, initial fluoroscopic assessment of the tip and catheter course, correction when indicated, and final fluoroscopic confirmation followed by postoperative chest radiography (Figure 1). Operators assessed tip depth and course fluoroscopically; a proximal or distal tip or an abnormal course prompted correction, with the maneuver selected according to the finding. Every catheter correction was performed by the operator under fluoroscopic guidance during the same procedure. First-pass target success referred to a satisfactory tip position and course before correction. The corrected record classified 224 first-pass failures as a proximal tip (118), distal tip (61), or abnormal catheter course (45). Advancement, withdrawal, guidewire-assisted repositioning, and other maneuvers were nonexclusive correction types. Intraprocedural catheter malposition was recorded as a separate finding. Eleven corrected cases had no specific maneuver code.

### 2.4. Imaging Measurements and Reference-Length Reconstruction

The carina served as the radiographic landmark. Guidance estimates the cavoatrial junction (CAJ) at approximately 4 cm below the carina, whereas a 2024 CT study reported a mean distance of 46.2 mm with patient-to-patient variation [1,11]. For this analysis, the institutional optimal band was 20–40 mm below the carina, with 10–20 mm and 40–45 mm as adjacent acceptable bands. These categories describe radiographic placement relative to the carina. The final target-success field denotes the optimal band; broader acceptability includes the two adjacent bands.

The imaging-defined reference length was reconstructed independently of the retained operative length by tracing the catheter route to the CAJ-oriented target region. Retained operative length was abstracted separately from the procedure record. Signed length error was defined as retained length minus imaging-defined reference length, and absolute length error as its absolute value. The imaging-defined reference served as a protocol-based geometric comparator, and model fit describes calibration within this cohort. Recent chest-radiograph prediction and three-dimensional reconstruction studies also show the importance of the selected anatomical target and imaging method in catheter-length planning [8,9].

### 2.5. Procedural and Clinical Outcomes

Procedural outcomes included first-pass target success, correction and coded maneuver type, repeated adjustment (at least two recorded adjustments), abnormal initial catheter course, recorded intraprocedural catheter malposition, and final tip category. Abnormal course included azygos entry or kinking. Immediate complications were pneumothorax, hemothorax, bleeding, and persistent immediate malposition. The 90-day first device-related event was categorized as mechanical, thrombotic, infectious, or other according to the adjudication record. Mechanical events comprised clinically consequential migration, nonthrombotic occlusion, kinking, dislodgment, port rotation, or fracture. Thrombotic events included clinically apparent or imaged thrombosis, tip thrombus, or fibrin sheath. Infectious events included microbiologically supported catheter-related bloodstream infection and port-pocket, tunnel, or exit-site infection. Other events were device-related clinical encounters outside these categories. Port-attributable chemotherapy delay was recorded separately and could overlap with an event.

The analysis used a 90-day window to describe early device events after implantation, matching the available structured follow-up. Patient-level first-event proportions use the 823 patients with documented 90-day status. Time to first event was calculated from implantation to its adjudicated date. For first-event incidence density, observation ended at the first event or recorded last contact, up to day 90; 13 patients without complete 90-day status contributed their observed time. The all-episode incidence density counts first and recurrent events over total documented 90-day catheter time, including time after a first event. The adjudication log recorded 13 recurrent episodes in addition to the 45 first events.

### 2.6. Imaging Displacement Threshold

The signed difference between the intraprocedural fluoroscopic and postoperative chest-radiograph tip positions was recorded in millimeters. The prespecified binary displacement field (>10 mm) agrees with the absolute numeric distance in the corrected workbook. Counts above 5, 10, and 15 mm are reported in Supplementary Table S1. The interval between images and independent paired repeat measurements were not recorded.

### 2.7. Statistical Analysis

Continuous-variable distributions were reviewed by histograms and Shapiro–Wilk tests. Approximately symmetric measures are reported as mean ± standard deviation and compared with Welch’s *t* test. Skewed puncture-attempt and fluoroscopy-time distributions are reported as median (interquartile range) and compared with the Mann–Whitney U test. Baseline characteristics remain descriptive. Binary outcomes are summarized as left-minus-right risk differences with Newcombe–Wilson 95% confidence intervals (CIs); selected *p* values use Fisher’s exact test. Immediate complications and event-category timing are descriptive. Center-stratified procedural counts and displacement thresholds appear in Supplementary Table S1; immediate complications and time to first event appear in Supplementary Table S2. An internal linear model related imaging-defined reference length to access side, height, body mass index, subcutaneous length (L2), and center. Adjusted R^2^ and apparent mean absolute error (MAE) describe fit within this cohort. Analyses were performed in Python 3.13.14.

## 3. Results

### 3.1. Cohort and Baseline Profile

The cohort included 836 women: 508 at Center A and 328 at Center B. Left IJV access was used in 478 procedures and right IJV access in 358. Under the contralateral access policy, right breast disease corresponded to left venous access and vice versa. Table 1 summarizes baseline and procedural features. All procedures used ultrasound-guided venipuncture and fluoroscopic positioning with the same 6.6-F port platform. Supplementary Table S1 provides center-stratified procedural counts.

### 3.2. Access-Side Imaging and Procedural Profile

Left-sided procedures followed a longer route. Mean retained catheter length was 24.5 ± 1.0 cm on the left and 22.2 ± 0.8 cm on the right; imaging-defined reference length was 25.0 ± 0.5 and 22.2 ± 0.5 cm. The intravascular component was approximately 2.0 cm longer on the left. Procedure duration and fluoroscopy time were similar between groups (Table 1).

**Figure 2:**
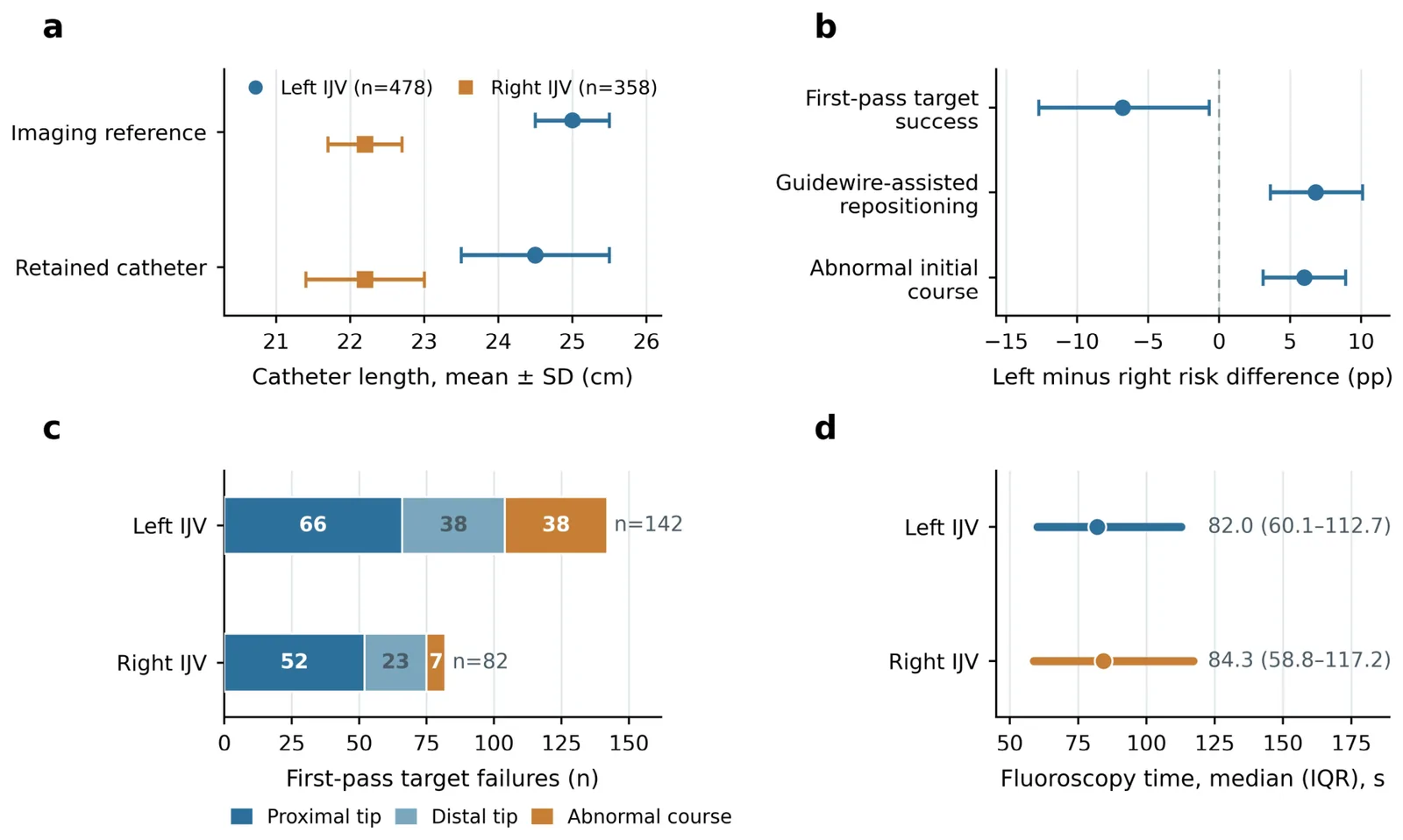
Four-panel summary of access-side geometry and fluoroscopic positioning. (**a**) Imaging-defined reference and retained catheter lengths (mean ± standard deviation). (**b**) Left-minus-right absolute risk differences with Newcombe–Wilson 95% CIs for first-pass target success, guidewire-assisted repositioning, and abnormal initial catheter course. (**c**) Mutually exclusive recorded reasons for the 224 first-pass target failures: proximal tip, distal tip, or abnormal course. (**d**) Fluoroscopy time shown as median and interquartile range. Exact numerators, denominators, and other procedural findings are in Table 1 and Table 2. CI, confidence interval; IJV, internal jugular vein; pp, percentage points.

First-pass target success was recorded in 336/478 (70.3%) left-sided and 276/358 (77.1%) right-sided procedures (left-minus-right risk difference, −6.8 percentage points; 95% CI, −12.7 to −0.7). Correction was required in 142/478 (29.7%) and 82/358 (22.9%). Guidewire-assisted repositioning was used in 46/478 (9.6%) and 10/358 (2.8%); an abnormal initial course was recorded in 38/478 (7.9%) and 7/358 (2.0%) (Figure 2; Table 2). The abnormal courses comprised 30 azygos entries, 11 kinks, and 4 cases with both findings. A separate intraprocedural malposition field was positive in 42 left-sided and 24 right-sided procedures. At each center, guidewire-assisted correction and abnormal initial course were more frequent on the left (Supplementary Table S1).

### 3.3. Final Tip Position and Reference-Length Calibration

The final optimal category included 464/478 (97.1%) left-sided and 346/358 (96.6%) right-sided tips. The adjacent acceptable categories contained 11 and 10 tips; thus, 475/478 (99.4%) and 356/358 (99.4%) were within the broader 10–45 mm band. Five final tips were outside that band. The internal reference-length model yielded adjusted R^2^ = 0.891, apparent MAE = 0.39 cm, and a left-sided coefficient of +2.82 cm (Supplementary Figure S1). Absolute tip-position differences > 5, >10, and >15 mm between fluoroscopy and postoperative radiography are reported in Supplementary Table S1.

### 3.4. Immediate Complications and Ninety-Day Events

Three pneumothoraces, all after left IJV access, and six bleeding events (four left, two right) were documented immediately after implantation. These nine events involved distinct patients; no hemothorax or persistent immediate malposition was recorded (Supplementary Table S2). Ninety-day status was available for 823/836 patients (98.4%): 471/478 left and 352/358 right. A first device-related event occurred in 27/471 (5.7%) and 18/352 (5.1%), respectively (risk difference, +0.62 percentage points; 95% CI, −2.71 to 3.72).

Adjudication identified 12 mechanical first events (11 left, 1 right), 18 thrombotic (11 left, 7 right), 10 infectious (4 left, 6 right), and 5 other (1 left, 4 right). Median time to the first event was 29 days (interquartile range, 18–41; range, 7–88). The 45 first events arose during 71,383 catheter-days accrued before the first event or last contact, yielding 0.63 first events per 1000 catheter-days. Thirteen recurrent episodes (11 mechanical and 2 thrombotic) occurred within 90 days for 58 recorded episodes during 73,908 catheter-days (0.78 episodes per 1000 catheter-days). Fifteen patients had a documented port-attributable chemotherapy delay (10 left, 5 right). Table 3 summarizes outcomes by access side. Supplementary Table S2 shows event timing, episode burden, and immediate-complication details.

**Figure 3:**
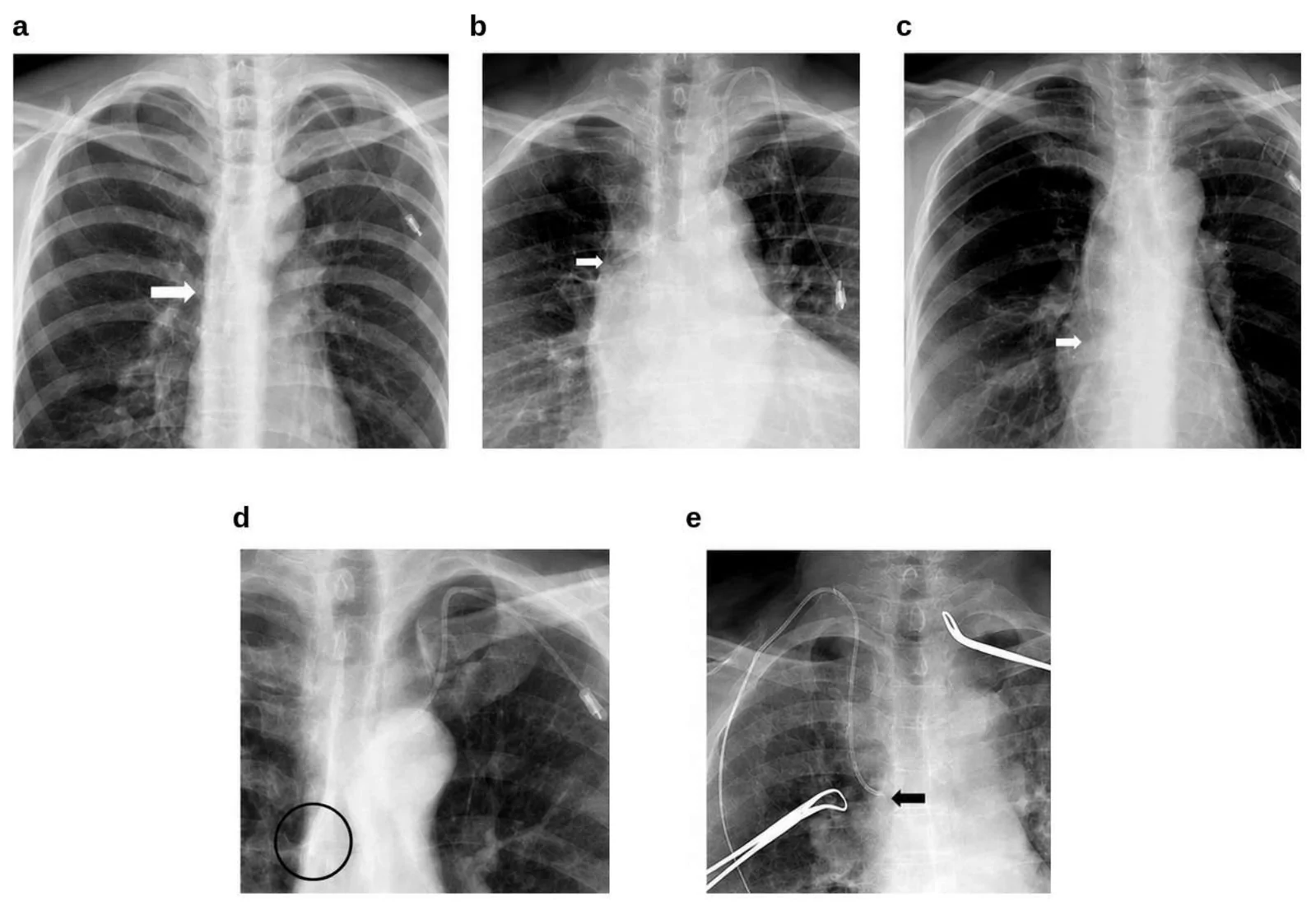
Representative anonymized images used for procedural and postoperative classification. (**a**) Catheter tip in the intended target region. (**b**) Proximal tip position. (**c**) Distal tip position. (**d**) Abnormal intraprocedural catheter course, illustrated by azygos entry or kinking and marked by the black circle. (**e**) Image-guided corrective repositioning in progress. The black arrow marks the abnormal position of the distal catheter tip during fluoroscopic adjustment. The panels illustrate different patients and are not a temporal sequence. White arrows in panels (**a**–**c**) indicate the catheter tips.

Representative procedural and postoperative images are shown in Figure 3. The examples illustrate target, proximal and distal tip positions, an abnormal catheter course, and image-guided corrective repositioning.

## 4. Discussion

Left IJV port placement in this cohort required a longer catheter route and more frequent correction of the initial course and tip position. Fluoroscopy displayed these findings during placement and guided correction. More than 96% of final tips were within the optimal 20–40 mm band on each side. Nine immediate complications and 45 first device-related events were recorded.

Fluoroscopy captured the initial catheter course, corrective maneuvers, and final tip during a single procedure. A radiologically guided series of 1180 oncology ports reported catheter malposition and migration among delayed complications [16]. A standardized right-IJV cohort used ultrasound-guided puncture, fluoroscopic tip positioning, and postoperative radiography to document procedural and clinical results [17]. Our side-specific data show which maneuvers were required between initial assessment and final fixation. Reviewing these sequences may help trainees recognize left-sided route geometry and discuss corrective choices during supervised procedures; educational outcomes were not measured.

The left-sided route crosses the brachiocephalic vein before entering the SVC. Wang et al. described DSA-guided adjustment of displaced or kinked ports [18]. In a predominantly breast-cancer cohort, Kim reported mean catheter lengths of 23.7 cm from the left IJV and 21.2 cm from the right under ultrasound and fluoroscopy guidance [19]. Our cohort showed a similar route-length difference, together with more guidewire-assisted repositioning and abnormal initial course on the left at both centers. Intraprocedural catheter malposition was analyzed separately from corrective maneuvers.

A 2026 right-IJV series described ultrasound-guided, fluoroscopy-free placement with height-based length estimation [20]. Park et al. assessed wall abutment under fluoroscopy and documented subsequent changes on CT [21]. In our left-IJV cases, fluoroscopy displayed the brachiocephalic-to-SVC turn, initial tip, and corrective maneuvers in real time. The effect of intraprocedural imaging on clinical outcomes warrants comparative study.

The 20–40 mm optimal band is an institutional radiographic convention. Current vascular-access guidance places the CAJ at approximately 4 cm below the carina [1], while CT reconstruction found a mean distance of 46.2 mm with interpatient variation [11]. Hamri et al. observed more complications with tips ≤ 10 mm below the carina [12]. A 2025 breast-cancer laterality study addressed side selection and clinical events in a smaller cohort [22], and a breast-cancer case series illustrated thrombosis with deeply situated right-atrial tips [23]. These findings support attention to the lower SVC–CAJ region, catheter trajectory, and avoidance of marked proximal or distal positioning.

The supporting length model summarizes the geometry of this cohort. Access side accounts for much of its internal fit; height, body mass index, L2, and center make smaller contributions. A 2024 implantation study highlighted catheter bending at the pocket-to-catheter junction as a technical consideration [24]. A 2026 right-IJV series used intracavitary ECG for tip localization [25]. In our workflow, reference length provided an initial estimate, and the operator confirmed catheter course and terminal tip under fluoroscopy.

The adjudication log contained 45 first events and 13 recurrent episodes during 90-day follow-up, with thrombotic, mechanical, infectious, and other event categories. We report first-event risk and total episode burden with their respective catheter-day denominators; immediate complications are tabulated separately.

The contralateral access policy linked venous access side to cancer laterality, and eight operators performed the procedures. Both centers used the same 6.6-F port platform and fluoroscopic guidance. The process measures describe procedural complexity; their association with later clinical outcomes requires further study. The 20–40 mm band was an institutional radiographic target rather than a patient-specific CAJ measurement. Eleven corrected procedures lacked a specific maneuver code. Longer follow-up and comparative studies across devices and guidance techniques would clarify clinical outcomes and generalizability.

## 5. Conclusions

Compared with right IJV access, left IJV placement required a longer catheter route and more guidewire-assisted repositioning. Operators identified and corrected abnormal initial courses under fluoroscopy; more than 96% of final tips on each side were in the institutional optimal band. First device-related events occurred in 45 of 823 patients with documented 90-day status.

## Figures and Tables

**Table 1:** Patient characteristics and procedural features according to IJV access side.

Characteristic	Left IJV (n = 478)	Right IJV (n = 358)	*p* Value
Center A	290 (60.7)	218 (60.9)	—
Center B	188 (39.3)	140 (39.1)	—
Age, years	51.5 ± 8.4	53.1 ± 9.8	—
Height, cm	160.5 ± 4.5	161.3 ± 4.6	—
Weight, kg	63.0 ± 7.7	63.5 ± 8.5	—
BMI, kg/m^2^	24.5 ± 3.3	24.5 ± 3.5	—
Prior breast/axillary surgery	425 (88.9)	333 (93.0)	—
Chemotherapy indication at implantation	478 (100.0)	358 (100.0)	—
Procedure and geometry			
Puncture attempts, median (IQR)	1 (1–1)	1 (1–1)	0.100
Guidewire first-pass success	408/478 (85.4)	319/358 (89.1)	0.120
Final retained catheter length, cm	24.5 ± 1.0	22.2 ± 0.8	<0.001
Subcutaneous component (L2), cm	6.2 ± 0.7	5.8 ± 0.7	<0.001
Imaging-defined reference length, cm	25.0 ± 0.5	22.2 ± 0.5	<0.001
Procedure time, min	23.7 ± 5.8	23.9 ± 6.4	0.618
Fluoroscopy time, s, median (IQR)	82.0 (60.1–112.7)	84.3 (58.8–117.2)	0.525

Values are mean ± SD, median (IQR), or n (%). Baseline characteristics are descriptive without hypothesis tests. Procedure-related *p* values are exploratory; Mann–Whitney U tests were used for puncture attempts and fluoroscopy time, Welch’s *t* tests for other continuous measures, and Fisher’s exact test for guidewire first-pass success. BMI, body mass index; IJV, internal jugular vein.

**Table 2:** Fluoroscopic positioning workflow and final target-zone achievement according to access side.

Metric	Left IJV (n = 478)	Right IJV (n = 358)	Risk Difference, pp (95% CI)
First-pass target success	336 (70.3)	276 (77.1)	−6.8 (−12.7 to −0.7)
Any corrective maneuver	142 (29.7)	82 (22.9)	+6.8 (0.7 to 12.7)
Catheter advancement	63 (13.2)	43 (12.0)	+1.2 (−3.5 to 5.6)
Catheter withdrawal	41 (8.6)	32 (8.9)	−0.4 (−4.4 to 3.5)
Guidewire-assisted repositioning	46 (9.6)	10 (2.8)	+6.8 (3.6 to 10.1)
Other coded maneuver	6 (1.3)	3 (0.8)	+0.4 (−1.3 to 2.0)
Repeated adjustment (≥2)	54 (11.3)	26 (7.3)	+4.0 (0.0 to 7.9)
Abnormal initial catheter course	38 (7.9)	7 (2.0)	+6.0 (3.1 to 8.9)
Intraprocedural catheter malposition	42 (8.8)	24 (6.7)	+2.1 (−1.7 to 5.7)
Final optimal zone, 20–40 mm	464 (97.1)	346 (96.6)	+0.4 (−2.0 to 3.1)
Final marginal proximal, 10–20 mm	10 (2.1)	7 (2.0)	—
Final marginal distal, 40–45 mm	1 (0.2)	3 (0.8)	—
Final within broader acceptable band, 10–45 mm	475 (99.4)	356 (99.4)	−0.1 (−1.3 to 1.4)
Final outside acceptable band	3 (0.6)	2 (0.6)	+0.1 (−1.4 to 1.3)
Image-to-image difference > 10 mm	11 (2.3)	15 (4.2)	−1.9 (−4.7 to 0.5)

Values are n (%) unless otherwise stated. Absolute differences are left-minus-right risk differences with Newcombe–Wilson 95% CIs. The final target-success field denotes the institutional optimal 20–40 mm category; 10–20 mm and 40–45 mm are adjacent acceptable categories. Correction maneuvers are nonexclusive. Intraprocedural catheter malposition is a finding rather than a correction maneuver. Eleven corrected cases have no coded maneuver type. CI, confidence interval; IJV, internal jugular vein; pp, percentage points.

**Table 3:** Immediate complications and 90-day device-related outcomes according to access side.

Outcome	Left IJV	Right IJV	Absolute Difference (95% CI)	*p* Value
Any immediate complication	7/478 (1.5)	2/358 (0.6)	—	—
Pneumothorax	3/478 (0.6)	0/358 (0.0)	—	—
Bleeding	4/478 (0.8)	2/358 (0.6)	—	—
90-day follow-up complete	471/478 (98.5)	352/358 (98.3)	+0.21 (−1.56 to 2.28) pp	0.787
Any 90-day device-related first event	27/471 (5.7)	18/352 (5.1)	+0.62 (−2.71 to 3.72) pp	0.758
Mechanical first event	11/471 (2.3)	1/352 (0.3)	—	—
Thrombotic first event	11/471 (2.3)	7/352 (2.0)	—	—
Infectious first event	4/471 (0.8)	6/352 (1.7)	—	—
Other first event	1/471 (0.2)	4/352 (1.1)	—	—
Recurrent device-event episodes, n	12	1	—	—
All device-event episodes, n	39	19	—	—
All-episode incidence per 1000 catheter-days	0.92	0.60	—	—
Port-attributable chemotherapy delay	10/471 (2.1)	5/352 (1.4)	+0.70 (−1.39 to 2.62) pp	0.601

Immediate-event denominators include all procedures; 90-day first-event and chemotherapy-delay denominators include patients with documented 90-day status. First-event categories are mutually exclusive. Recurrent and all-episode rows count episodes; a patient may contribute more than one event. Episode incidence density uses all documented 90-day catheter-days, whereas first-event density censors at the first event. Category rows and immediate complications are descriptive. Chemotherapy delay can overlap with a device event. The *p* values for follow-up completeness, any first event, and chemotherapy delay are two-sided Fisher’s exact tests. CI, confidence interval; IJV, internal jugular vein; pp, percentage points.

## Data Availability

The datasets generated and/or analyzed during the current study are available from the corresponding author on reasonable request, subject to institutional and ethical requirements.

## Supplementary Materials

The following supporting information can be downloaded at: https://www.mdpi.com/article/10.3390/diagnostics16203310/s1, Figure S1: Internal reference-length calibration; Table S1: Center-stratified positioning and image displacement; Table S2: Immediate complications and 90-day event timing.

## Abbreviations

BMI, body mass index; CAJ, cavoatrial junction; CI, confidence interval; CXR, chest radiograph; DSA, digital subtraction angiography; IJV, internal jugular vein; MAE, mean absolute error; SVC, superior vena cava; TIVAD, totally implantable venous access device.
