# Supplementary material for: Fluoroscopic Catheter-Tip Positioning During Internal Jugular TIVAD Placement in Breast Cancer: A Two-Center Retrospective Cohort Study
Source: Diagnostics (Basel). 2026 Oct 12;16(20):3310. doi: 10.3390/diagnostics16203310 (PMC13702922; doi:10.3390/diagnostics16203310)
Supplement: Supplementary file 1 [file diagnostics-16-03310-s001.zip › diagnostics-4579943-supplementary.pdf]

Figure S1. Internal reference-length calibration

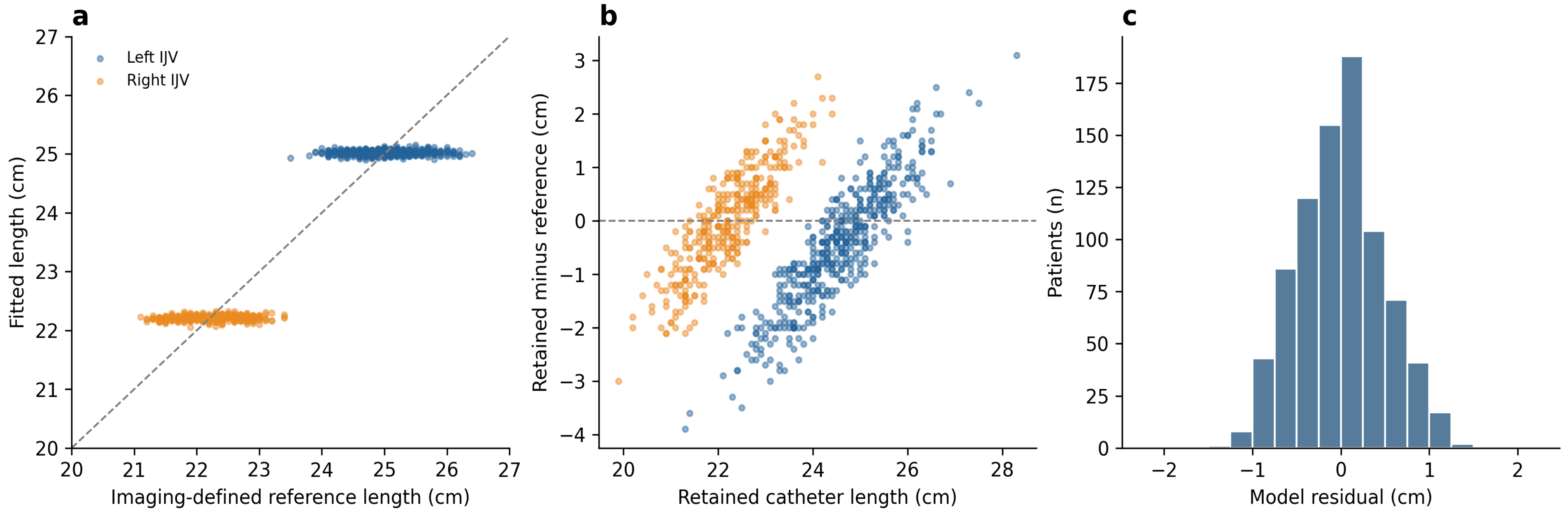

(a) Fitted versus imaging-defined reference length, by access side; dashed line indicates equality. (b) Retained minus reference length by retained catheter length. (c) Distribution of model residuals.

Internal fit: adjusted $R^2 = 0.891$; apparent mean absolute error = 0.39 cm. The model included access side, height, body mass index, subcutaneous length (L2), and center. It describes geometry within this cohort; external validation was not performed.

Table S1. Center-stratified positioning and image displacement

Center and access side	n	First-pass success	Any correction	Guidewire-assisted repositioning	Abnormal initial course
Center A, left IJV	290	200/290 (69.0%)	90/290 (31.0%)	32/290 (11.0%)	25/290 (8.6%)
Center A, right IJV	218	162/218 (74.3%)	56/218 (25.7%)	4/218 (1.8%)	3/218 (1.4%)
Center B, left IJV	188	136/188 (72.3%)	52/188 (27.7%)	14/188 (7.4%)	13/188 (6.9%)
Center B, right IJV	140	114/140 (81.4%)	26/140 (18.6%)	6/140 (4.3%)	4/140 (2.9%)

Counts use all procedures in each center and access-side stratum. Corrective maneuvers can overlap.

Absolute fluoroscopy-to-radiograph tip difference	Left (n = 478)	Right (n = 358)	Overall (n = 836)
>5 mm	98	106	204
>10 mm	11	15	26
>15 mm	0	0	0

The recorded >10 mm displacement flag agreed with the strict threshold applied to the numeric measurements in all 836 cases.

Table S2. Immediate complications and 90-day event timing

Immediate complication	Overall	Left IJV	Right IJV	Center A	Center B
Pneumothorax	3	3	0	2	1
Hemothorax	0	0	0	0	0
Bleeding	6	4	2	5	1
Persistent immediate malposition	0	0	0	0	0

The three pneumothoraces and six bleeding events involved nine distinct patients.

First-event category	n	Median (days)	IQR (days)	Range (days)	After day 30, n
All first events	45	29	18–41	7–88	21
Mechanical	12	28.5	15.5–34.75	9–45	6
Thrombotic	18	25	17.25–28.75	7–55	3
Infectious	10	50.5	39.25–67.75	16–88	9
Other	5	31	25–47	19–48	3

First-event dates were concordant between the 90-day follow-up and event-adjudication records. After correction of the port-removal date in the source record, all 13 recurrent episodes occurred within 90 days and before port removal.

Episode burden during the 90-day observation window

First events: 45/71,383 first-event risk-days = 0.63 per 1000 catheter-days. Recurrent episodes: 13 (12 left, one right). All episodes: 58/73,908 catheter-days = 0.78 per 1000 catheter-days.

All-episode incidence by access side: left, 39/42,355 = 0.92; right, 19/31,553 = 0.60 per 1000 catheter-days.
